# Selenium-ruthenium complex blocks H1N1 influenza virus-induced cell damage by activating GPx1/TrxR1

**DOI:** 10.7150/thno.83522

**Published:** 2023-03-21

**Authors:** Yinghua Li, Danyang Chen, Jingyao Su, Mingkai Chen, Tianfeng Chen, Wei Jia, Bing Zhu

**Affiliations:** 1 Center Laboratory, Guangzhou Women and Children's Medical Center, Guangzhou Medical University, Guangzhou 510120, China; 2 Department of Chemistry, Jinan University, Guangzhou 510632, China; 3 Department of Pediatric Urology, Guangzhou Women and Children's Medical Center, Guangzhou Medical University, Guangzhou 510120, China

**Keywords:** ruthenium-selenium complexes, H1N1, ROS, selenoprotein, apoptosis

## Abstract

**Background:** Influenza A (H1N1) virus is an acute respiratory infectious disease that causes massive morbidity and mortality worldwide. As an essential trace element, selenium is widely applied in the treatment of various diseases because of its functions of enhancing immune response, antioxidant and antiviral mutation. In this study, we constructed the selenium-containing metal complex drug delivery system Ru(biim)(PhenSe)_2_ (**RuSe**), and investigated the anti-influenza virus efficacy and the potential antiviral mechanism for **RuSe**.

**Methods:** The inhibitory effect of **RuSe** on influenza-mediated apoptosis was examined by cell count assay, cell cycle assay, Annenxin-V assay, TUNEL-DAPI assay and reactive oxygen species level determination. Virulence assay, PCR and neuraminidase inhibition assay revealed the inhibition of **RuSe** on influenza virus. At the level of animal experiments, two animal models were used to clarify the role of **RuSe** through HE staining, immunohistochemical staining, cytokine determination, selenium metabolism determination and selenium protein expression level determination.

**Results:** The results of this study confirm that **RuSe** enhances the expression levels of selenium proteins GPx1 and TrxR1 by regulating selenium metabolism, thereby inhibiting viral replication and assembly and regulating virus-mediated mitochondria-related apoptosis. On the other hand, animal experiments show that **RuSe** can reduce lung tissue inflammation and inhibit lung tissue cell apoptosis in mice, and improve the survival state of mice. In addition, **RuSe** significantly improves the low immune response of Se-deficient mice by regulating selenium metabolism, and effectively alleviated lung fibrosis and lung tissue apoptosis in Se-deficient mice.

**Conclusions:** This study suggests that **RuSe** provides a promising new approach for the clinical treatment of influenza virus.

## Introduction

Influenza is an acute infectious disease caused by Influenza virus (IVs) infection of the respiratory tract, with strong pathogenicity, rapid transmission, high morbidity and mortality 1. At present, the clinical treatment of influenza is mainly vaccination prevention and anti-influenza drug therapy 2. Due to the high variability of influenza virus and virus resistance to drugs, the application of conventional treatment is limited 3. Therefore, the development of efficient and broad-spectrum anti-influenza drugs has important clinical application value 4.

Selenium is an essential trace element with low toxicity and high biochemical activity 5, 6. Selenium deficiency can change the redox balance, weaken the host's antioxidant capacity and immune response, and lead to the increase of reactive oxygen species (ROS), thus promoting viral replication and reducing the body's antiviral immunity 7, 8. Sodium selenite and seleniumcontaining yeast (the main component of which is selenomethionine) are widely used as selenium sources to supplement selenium deficiency 9. However, the current selenium supplements are generally faced with low bioavailability, high toxicity, narrow safety range and other problems, which limit the application of selenium supplements 10. Metal complex drug delivery system has attracted the interest of a wide range of researchers due to their advantages of improving drug bioavailability and efficacy. In our previous studies, we found that introducing selenium atoms into metal complex drug delivery systems can significantly enhance biosafety 11, specifically inhibit TrxR activity 12, sensitize radiotherapy 13, 14, and enhance NK cell-mediated killing effect 15. Therefore, we are also interested in designing metal complexes as selenium-containing drug carriers to improve the availability of selenium and enhance the antiviral effect.

**Scheme 1:**
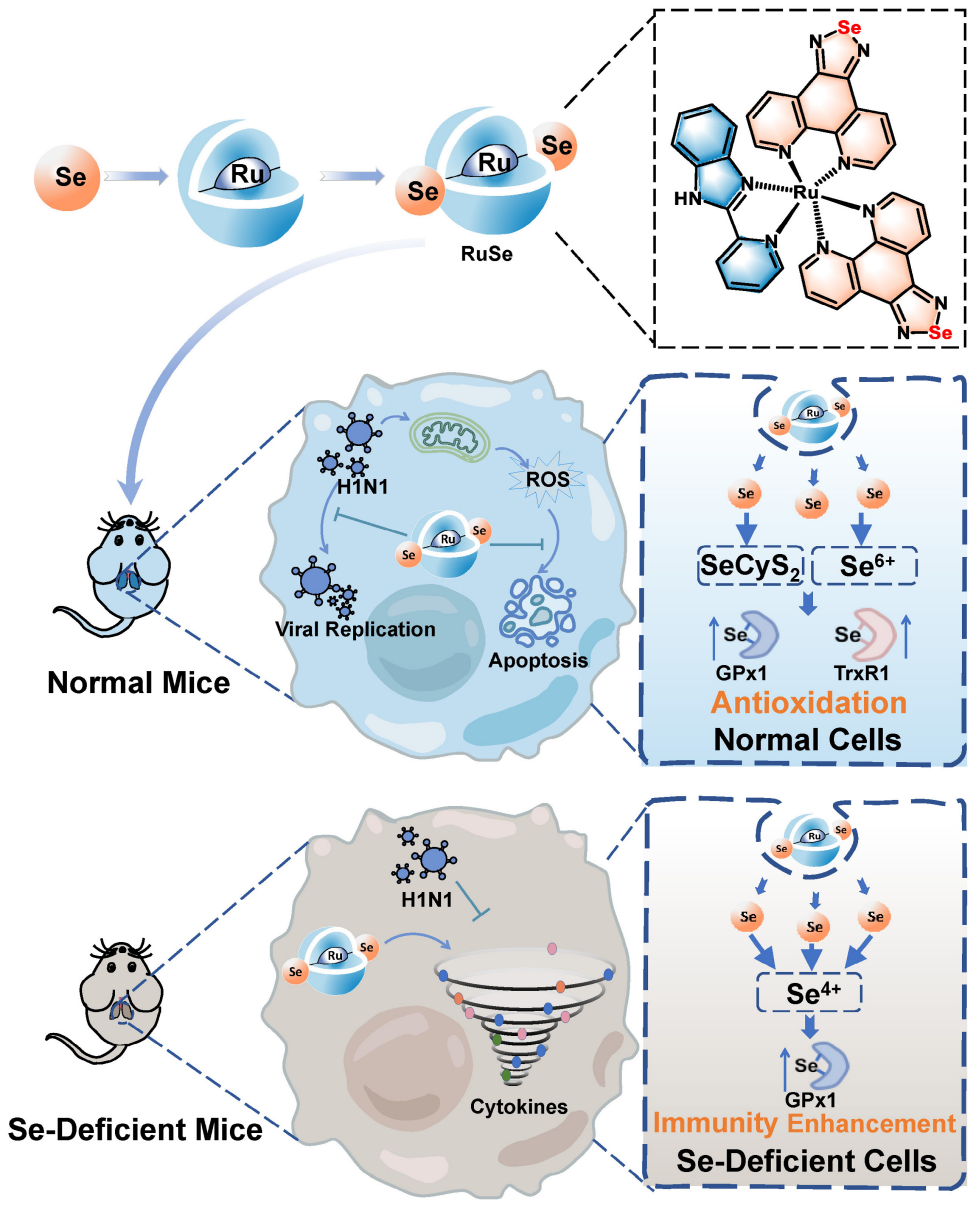
Schematic diagram of different inhibitory mechanisms of **RuSe** on pneumonia and apoptosis caused by H1N1 infection in two mouse models (normal mice and selenium-deficient mice).

Metal complexes have more excited electron configurations, coordination numbers and geometries, and thus exhibit excellent photochemical properties, high biological activity and enhanced design, and are widely used as diagnostic probes and therapeutic agents for diseases 16. At present, the antiviral research of complexes mainly focuses on Au (I), Pt (II) and Ag (I), Cu and metal-based nanomaterials, which have extensive antiviral activity, lasting and high efficiency 17. Currently, antiviral metal complexes are generally characterized by poor selectivity to normal cells, strong toxicity and enhanced chemical resistance 18. Therefore, it is urgent to develop new antiviral metal complexes, among which ruthenium metal complexes have low toxicity, strong binding ability to DNA, and rich electrochemical, photochemical, and photophysical properties 19. As shown in Scheme 1, we use selenium-containing 2-selenicimidazole[4,5-f]1,10-phenanthroline (PhenSe) as a selenium-containing ligand to construct Ru(biim)(PhenSe)_2_ (**RuSe**) by coordination with the co-ligand biimidazole as our previous study 15. As an anti-influenza drug,** RuSe** plays an antiviral role and also acts as a drug carrier to deliver selenium to the organism, regulate the selenium proteins GPx1 and TrxR1 *in vivo*, and play an antioxidant role in inhibiting ROS-mediated apoptosis. The results suggest that **RuSe** inhibits viral replication and assembly as well as regulate virus-mediated mitochondria-related apoptosis by regulating selenium metabolism and enhancing selenium protein activity. On the other hand, animal experiments show that **RuSe** can inhibit lung tissue inflammation and lung tissue apoptosis in mice, as well as improve the survival state of mice. In addition, in Se-deficient mouse models, **RuSe** significantly improved the low immune response caused by the viral infection and protected Se-deficient mice. In conclusion, this study suggests that the selenium-ruthenium complex may be an effective treatment strategy for influenza pneumonia.

## Materials and Methods

### Materials

MDCK cells used in the experiments were purchased from ATCC (Product code: ATCC CCL-34TM). H1N1 influenza virus was isolated and cultured from respiratory samples of patients with positive results of influenza A, which were provided by Guangzhou Women and Children's Medical Center, Guangzhou Medical University. Reagent for cell culture, including High-glucose DMEM, FBS, trypsin-EDTA (0.25%) and penicillin-streptomycin solution, were sourced from Gibco. Cell counting kit-8 (CCK-8), Cell cycle detection Kit, Annexin-V-Propidium iodide (PI) Co-staining kit, Neuraminidase activity (NA) detection kit, JC-1 mitochondrial membrane potential assay kit and the enhanced chemiluminescence (ECL) assay kit were purchased from Biotime Biotechnology. TPCK trypsin, 2',7'-dichlorofluorescein diacetate (DCF-DA), Tunel, and 4',6-diamidino-2-phenylindole (DAPI) were purchased from Sigma-Aldrich. Nucleoprotein (NP), hemagglutinin (HA), matrix protein 1 (M1), nonstructural protein 1 (NS1) and ion channel protein (M2) antibody were purchased from Abcam Reagent Co. The reverse transcription PCR kit was purchased from TAKARA Bio, Japan. The antibodies of Akt, p-Akt, PARP, cleaved PARP, Caspase-3, cleaved Caspase-3, p38, p-p38, STAT3, p-STAT3, Bcl-2 and Bax were purchased form Cell Signaling Technology (CST). Glutathione peroxidase (GPx1) and thioredoxin reductase (TrxR1) ELISA kits were purchased from Assay Genie Reagents.

### Cell culture and virus infection

Consistent MDCK cell culture methods, influenza H1N1 virus amplification methods and virulence measurement methods were consistent with our previous study 20. The TCID_50_ (median tissue culture infective dose) of H1N1 was calculated by Reed-Muench method when the cytopathic effect was stable. Once the H1N1 virus titer was determined, H1N1 virus with a titer of 100 TCID_50_/0.1 mL was used for *in vitro* studies in subsequent experiments.

### Synthesis

**PhenSe.** PhenSe was synthesized according to our previous methods 21.

**Ru(biim)(PhenSe)_2_** (**RuSe**). **RuSe** was synthesized according to our previous methods 12. Briefly, PhenSe (2 mmol, 0.57 g for PhenSe) were added to DMF solution of RuCl3·3H_2_O (1 mmol, 0.27 g) under argon atmosphere for 6 h at 140 °C. The precipitate was collected by filtration, 1 equivalent of 2-(2-pyridyl) benzimidazole was added to the solvent mixture (2-methoxyethanol: H_2_O = 3:1) and reflux was continued at 110 °C for 6 h. After the addition of NaClO_4_, the precipitate was collected and purified by alumina column chromatography to obtain **RuSe**.

### Cell viability test

CCK-8 assay was used to detect the cytotoxicity and then the antiviral effect of **RuSe**. In brief, **RuSe** was diluted in medium to the following concentrations: 16 μM, 8 μM, 4 μM, 2 μM, and 1 μM and added to the cells for 48 h to observe the cytotoxicity. The antiviral effect of **RuSe** was determined by setting the same range. The cytotoxicity and antiviral effect of **RuSe** were calculated based on the cell viability.

### Cell cycle assay

Four MDCK cell groups were set respectively, which were seeded in 6 cm Petri dishes: normal control group, H1N1 infection group, **RuSe** control group, H1N1 virus infection and **RuSe** treatment group (H1N1+**RuSe**). After different operations, the cells in each group were cultured in 33°C, 5% CO_2_ incubator for 48 h, and then digested by trypsin into a centrifuge tube. After that the cells were collected by centrifugation. After the collected cells were fixed and washed, RNaseA was added to remove RNA interference, and then PI (propidium iodide) was added and incubated in a dark room for 30 min. Fluorescence content was measured using flow cytometry with at least 20 000 cells per independent sample. The cycle distribution of cell populations was analyzed as previously described 22.

### Annexin-V/PI co-staining assay

MDCK cells infected with H1N1 and treated with **RuSe** were collected, fluorescence-dye-labeled Annexin-V and PI were added to the cells according to the instructions and incubated in the dark. Fluorescence of adherent cells was photographed using a Leica inverted fluorescence microscope, and fluorescence of suspended cells was determined using flow cytometry.

### RT-PCR

Relative RNA levels of H1N1 virus were measured using RT-PCR. RNA was extracted according to the tRNA extraction manual, and then the viral nucleoprotein NP was amplified by RT-PCR kit. The sequences of the 2 primer pairs were as follows: H1N1 forward primer 5' CTCAGCAAATCCTACATTA 3' and reverse primer 5' TAGTAGATGGATGGTGAAT 3'. GAPDH forward primer 5' CGCCAAGAAGGTGATCATTTC 3', and reverse primer 5' CAGGAGGCGTTCGAGATGAC 3'.

### Neuraminidase inhibition assay

The effect of **RuSe** on the activity of influenza virus neuraminidase was detected by Neuraminidase Assay Kit (Applied Beyotime, CHINA). The **RuSe** was serially diluted and mixed with influenza virus, and the NA activity of influenza virus was detected by neuraminidase detection kit. NA assay results are presented as mean ± SEM of three independent experiments (n=3).

### Electron microscopic morphology observation

MDCK cells of different treatments were collected, and used 2.5% glutaraldehyde to fixed cellular morphology for 2 h, then centrifuged to discard the supernatant. After drying, dewatering, embedding, sectioning and negative staining, the cells were observed under electron microscope.

### Immunofluorescence detection of NP in influenza

Cells were seeded in wells with partitions and divided into 4 groups: Control group, H1N1 group, **RuSe** group and H1N1+**RuSe** group, which were given corresponding treatment. After 24 h, cells were fixed and membranes were broken with Triton X-100, then MDCK were blocked with 1% BSA for 30 min. Anti-NP antibody was added to the cells and incubated for 1h. After washing, Alexa Fluor®488 conjugated antibody was added to bind with primary antibody. The blocking agent was dropped onto glass slides, and the expression of NP was observed by fluorescence microscopy.

### Western blot

MDCK cells were seeded in 10 cm culture dish at a concentration of 8*10^4^/mL, respectively. When the cell density reached 80%-90%, the cells in each group were treated with corresponding operations and cultured for 48 h. Lysis, denaturation, electrophoretic separation and western blotting of total intracellular proteins were performed as described in previous studies. The primary antibodies were incubated overnight, then the secondary antibody was incubated and the image were photographed by the Thermo Fisher Gel Imaging system 22.

### Mitochondrial membrane potential assay

One of the symptoms of apoptosis is an imbalance of mitochondrial membrane potential. MDCK cells were seeded in 24-well plates and treated with 8 μM **RuSe** for 48 h after virus infection. Leica fluorescence microscope was used to record the mitochondrial membrane potential of adherent cells and flow cytometry was used to detect the suspended cells.

### Determination of active-oxygen content

The non-fluorescent substance DCFH-DA can be oxidized by intracellular free radicals to produce DCF, which can emit green fluorescence. Therefore, the effect of **RuSe** on the intracellular ROS (reactive oxygen species) in MDCK cells could be detected by DCFH-DA. MDCK infected with H1N1 was treated with **RuSe** for 48 h, followed by incubation with DCFH-DA at a concentration of 10 μM for 20 min. And then the fluorescence intensity was recorded with a Leica fluorescence microscope 23.

### TUNEL-DAPI co-staining assay

The cell apoptosis was detected using the TUNEL-DAPI co-staining kit as previously reported 24. Briefly, MDCK cells infected with H1N1 were treated with a concentration of 8 μM **RuSe** for 48 h. The cells were detected by TUNEL-DAPI apoptosis kit. Intracellular DNA damage was photographed by Leica inverted fluorescence microscope.

### Cytokine detection

Cytokines are markers of inflammation in mice infected with H1N1. The serum of each group of mice was collected and the content of cytokines in serum was determined by multi-factor flow cytometry.

### Animal infection and treatment

Female BALB/c mice aged 4-6 weeks were randomly divided into groups with 6 mice in each group. Mice were weighed and injected with 10% chloral hydrate at a standard of 3 μL/g. Then, both normal control and drug control mice were given 20 μL saline by intranasal drip while the H1N1 group and H1N1 + **RuSe** group were dripped with 20 μL H1N1 diluted solution at a titer of 1000 TCID_50_/0.1 mL. 24 h later, mice in H1N1 group and H1N1+**RuSe** group were given nasal drip with **RuSe** at a concentration of 2.5 mM for three consecutive days 25. The body weight were recorded daily, and the clinical symptoms of mice were observed. After 14 days, the mice were anesthetized with chloral hydrate and the cervical vertebra was dislocated and the eyeball blood was collected. Mice were perfused with normal saline and fixed with paraformaldehyde for lung extraction. The lung tissue was weighed to calculate the lung index. Lung index is the percentage of lung mass to body mass, which usually indicates the severity of pneumonia. Then, the lung tissues of mice were embedded in paraffin and sectionalized for HE staining, TUNEl-DAPI assay and immunohistochemistry staining.

To further study the effects of selenium intake on mice infected with influenza, mice were fed with selenium deficiency. After feeding to the third generation, 4-6-week-old mice were infected with H1N1 virus, and each mouse was infected with 1000 TCID_50_/0.1 mL. After 1 day of infection, mice were treated with **RuSe** for 3 consecutive days at 50 mmoL/day. The mice were weighed daily. On the 14th day, the mice were sacrificed, the lung index was calculated, and lung tissue staining and immunohistochemistry were performed. The serum of mice was taken to determine cytokines.

### Determination of selenium content in lung tissue and serum of mice

0.5 g of lung tissue and 100 μL of serum were taken from mice and digested with 5 mL of mixed acid (perchloric acid: nitric acid = 1:3) overnight. Then the samples were heated on a heating plate until the solution becomes transparent. Add 3 mL HCL to the cooled solution and continue heating until the solution becomes transparent. After that, selenium content in lung tissue and serum of mice was measured by atomic fluorescence spectrometer.

### Determination of selenoenzymes GPx1 and TrxR1

The concentrations of selenoenzymes GPx1 and TrxR1 in 100 μL serum of mice were determined by ELISA. Detailed operation steps are carried out according to the instruction of ELISE GENIE (MOFI01171).

### Metabolism analysis of RuSe *in vivo*

The metabolism of **RuSe** in mice was examined by HPLC (Waters, 2695) coupled to inductive plasma mass spectrometry (PerkinElmer, 350x) (HPLC-ICP-MS, Hamilton PRP X-100 anion-exchange column). Selenium metabolites were quantified using standard samples (SeCyS_2_, SeCyS, SeIV, SeMet, and SeVI). Briefly, 100 μL of serum was taken from each mouse to quantify **RuSe** metabolites. The mobile phase detected was 10 mM citric acid solution (pH 4.5) at a flow rate of 1.5 mL/min.

### Statistical analysis

All experiments were carried out in triplicate and data were expressed as mean ± standard deviation (SD). Difference between the esanalyzed by two-tailed Student's t-test. *P* < 0.05 (*) or *P* < 0.01(**) were considered statistically significant.

## Results and Discussion

### RuSe significantly inhibits MDCK cells apoptosis induced by H1N1

**Figure 1:**
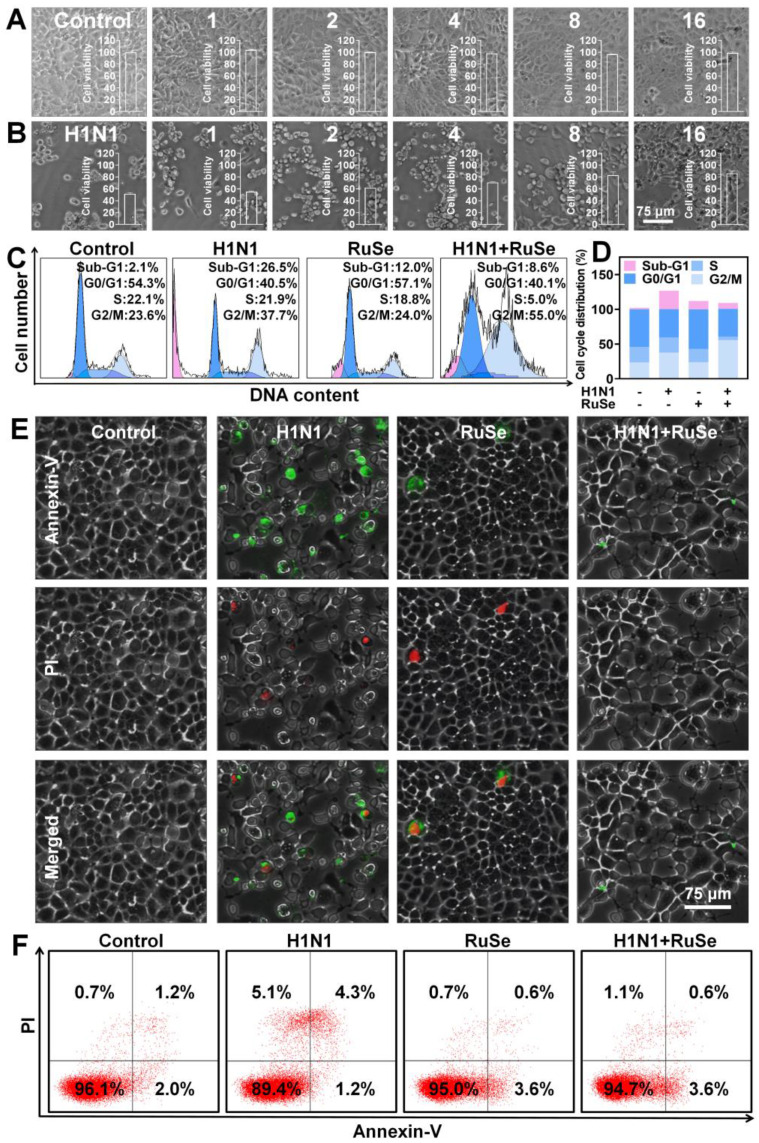
**RuSe inhibited influenza virus-mediated apoptosis.** (A) Cytotoxicity of **RuSe** at the concentrations of 1 μM, 2 μM, 4 μM, 8 μM, and 16 μM on MDCK cells. (B) Therapeutic effect of **RuSe** on H1N1-infected MDCK cells at concentrations of 1 μM, 2 μM, 4 μM, 8 μM, and 16 μM. (C and D) Effect of **RuSe** on apoptosis of MDCK cells mediated by H1N1 when **RuSe** concentration was 8 μM. (E) The apoptosis of MDCK cells infected with H1N1 was ob-served under fluorescence microscope using Annenxin-V and PI double staining. (F) The level of apoptosis of H1N1-infected MDCK cells was detected by flow cytometry using Annenxin-V and PI double staining. Each value represents means ± SD (n = 3).

Since safety issues largely determine the biomedical applications of selenium-ruthenium complexes, we first evaluated the *in vitro* safety of **RuSe**. As shown in Figure 1A, in the concentration range of 1 µM, 2 µM, 4 µM, 8 µM, 16 µM, **RuSe** showed little cytotoxicity. The results of CCK-8 were consistent with those observed under microscope. To investigate the antiviral activity of **RuSe**, we treated MDCK cells infected with H1N1 with **RuSe**. As shown in Figure 1B, the results showed that the cell activity of the H1N1 infected group was 51.6%, and that of the **RuSe** group was increased by 82.9% with 8 µM **RuSe** and 86.5% with 16 µM **RuSe**, which were significantly different from that of the H1N1 group. These results indicate that **RuSe** is nontoxic at certain concentrations and has certain therapeutic ability. On the other hand, it has been observed under inverted light microscope (Figure 1B) that the H1N1 infected cells showed typical morphological characteristics of apotosis, including overall cell roundness, cell pyknosis and reduced intercellular connections.

Influenza infection usually causes cell apoptosis. From the cell morphology under the microscope in Figure 1B, we speculated that **RuSe** inhibited cell apoptosis mediated by H1N1. To test this conjecture, we measured the cycle distribution of four groups of MDCK cells using a cell cycle test box. As shown in Figure 1C, the sub-G1 peak represents the proportion of apoptotic cells. The apoptosis rate increased to 26.5% after H1N1 infection, but decreased to 8.6% after **RuSe** treatment, indicating that **RuSe** inhibited the apoptosis of MDCK cells mediated by H1N1. Quantification of cell cycle distribution in each group is shown in Figure 1D. Annexin-V assay was further used to study cell membrane phosphatidylserine ectropy induced by H1N1 in order to reconfirm the inhibitory effect of **RuSe** on apoptosis. As shown in Figure 1E, the H1N1 group showed obvious phosphatidylserine translocation. After combined treatment with **RuSe**, the cell morphology returned to normal, and the phosphatidylserine ectropion was reduced. As shown in Figure 1F, Annexin-V and PI double positive represent late apoptosis. The apoptosis rate of cells in the H1N1 infection group was 4.3%, and that in the H1N1+ **RuSe** group was 0.6%. These results indicated that **RuSe** inhibited cell apoptosis induced by H1N1, which was consistent with the microscopic observation.

### RuSe inhibits replication and proliferation of influenza virus by inhibiting NP nuclear export

**Figure 2:**
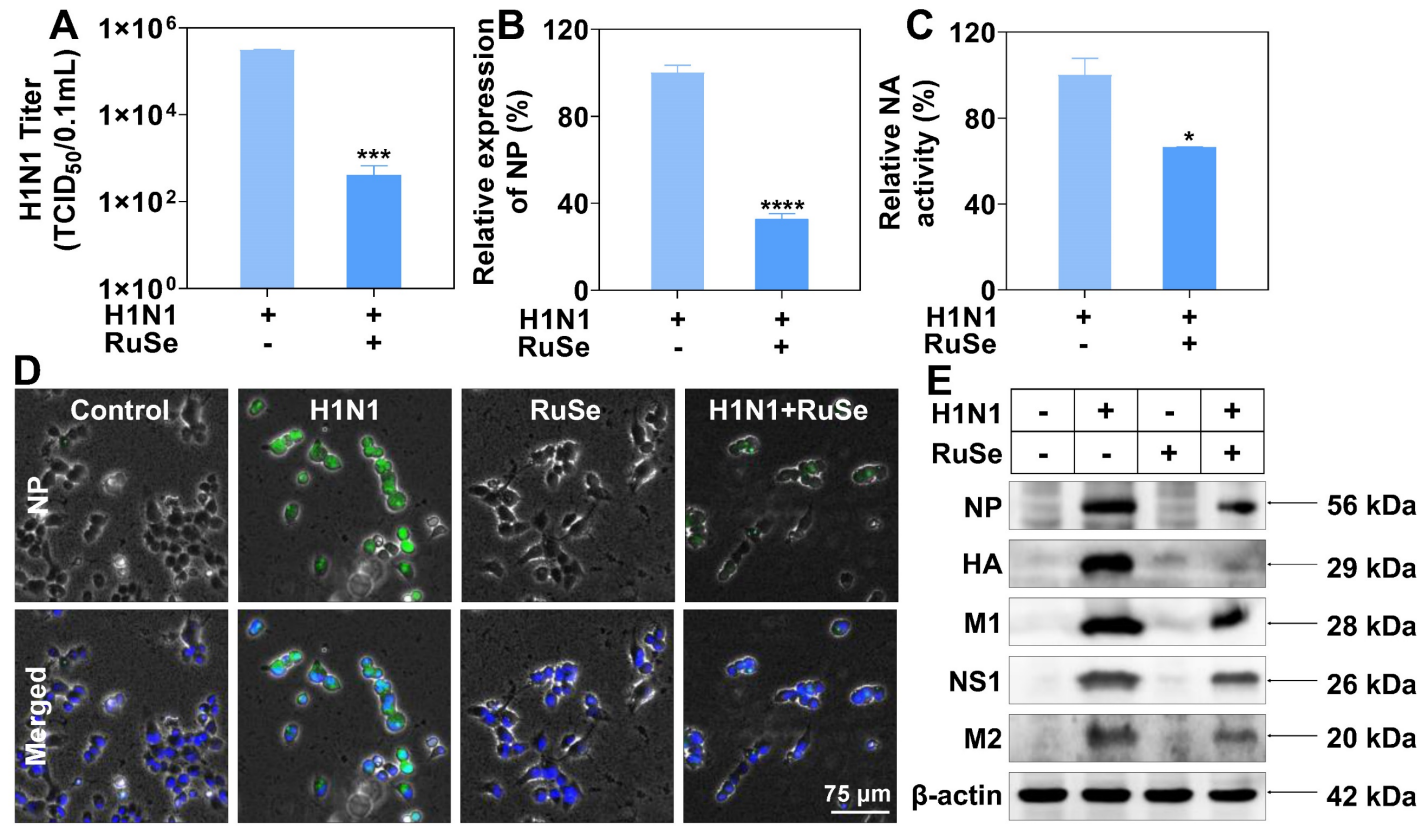
** RuSe inhibited the replication and proliferation of influenza virus by inhibiting NP nuclear export.** (A) Virulence assay (TCID50) on the supernatant of H1N1-infected and **RuSe** treated infected cells. (B) Comparison of H1N1 expression by Q-PCR between H1N1-infected and **RuSe**-treated groups. (C) NA activity in supernatants from H1N1 infected and **RuSe** treated groups was compared using a neuraminidase activity kit. (D) Immunofluorescence was used to detect the expression level and nuclear localization of influenza NP. The scale in the figure is 75 μm. (E) The inhibitory effect of **RuSe** on influenza NP, HA, M1, NS1 and M2 proteins was detected by Western blot. Each value represents means ± SD (n = 3).

Combined with the above results, **RuSe** can inhibit H1N1-mediated apoptosis. Whether the inhibitory effect of **RuSe** on apoptosis is related to the direct inhibitory effect of **RuSe** on influenza virus needs to be further explored. We then discussed the specific therapeutic effects of **RuSe**, and found that **RuSe** had significant inhibitory effects on virulence of H1N1 (Figure 2A), replication of viral nucleoprotein (NP) nucleic acid (Figure 2B) and activity of viral neuraminidase activity (NA) (Figure 2C). The inhibition of **RuSe** on virus virulence was determined by measuring the virulence of the progeny virus of the H1N1 group and the H1N1+ **RuSe** group (TCID_50_). The virulence of the progeny virus in the H1N1 group was 3.09*10^5^/0.1 mL, while that in the H1N1+ **RuSe** group was 1.04*10^2^/0.1 mL. The virulence of the progeny virus of the treatment group decreased significantly. At the same time, the determination of nucleoprotein (NP) of influenza virus showed that the relative NP expression of H1N1+**RuSe** group was 32.6% of the H1N1 group. In addition, the relative neuraminidase activity of the H1N1+ **RuSe** group was 66.3%.

**RuSe** does have a certain inhibitory effect on H1N1, but its mechanism has not been revealed. Influenza virus NP (nuclear protein) is the skeleton structure of vRNP (ribonucleoprotein particle), which plays an important role in the transcription and replication of the viral genome. Inhibition of nucleoplasmic shuttling of influenza virus NP is an effective method for the design of anti-influenza drugs. The screening of key targets of nucleoplasmic shuttling and the development of corresponding drugs are of great significance. As shown in Figure 2D, NP expression in the H1N1 infection group was high and mostly distributed in the cytoplasm. After addition of **RuSe** treatment, NP expression was inhibited in the nucleus. It can be seen that **RuSe** may inhibit viral replication and proliferation by inhibiting the nucleation of viral NP. As shown in Figure 2E, influenza virus-related proteins such as NP (nuclear protein), HA (hemagglutinin), M1 (matrix protein 1), NS1 (non-structural protein 1) and M2 (ion channel membrane protein 2) in the H1N1 infected group were all highly expressed in MDCK cells. After **RuSe** treatment, influenza virus-related proteins showed corresponding decrease, indicating that **RuSe** played an overall inhibitory effect on influenza virus replication and protein expression. Combined with the above results, **RuSe** can inhibit the replication and virulence of H1N1.

### RuSe inhibits cell apoptosis induced by mitochondrial oxidative damage mediated by H1N1

**Figure 3:**
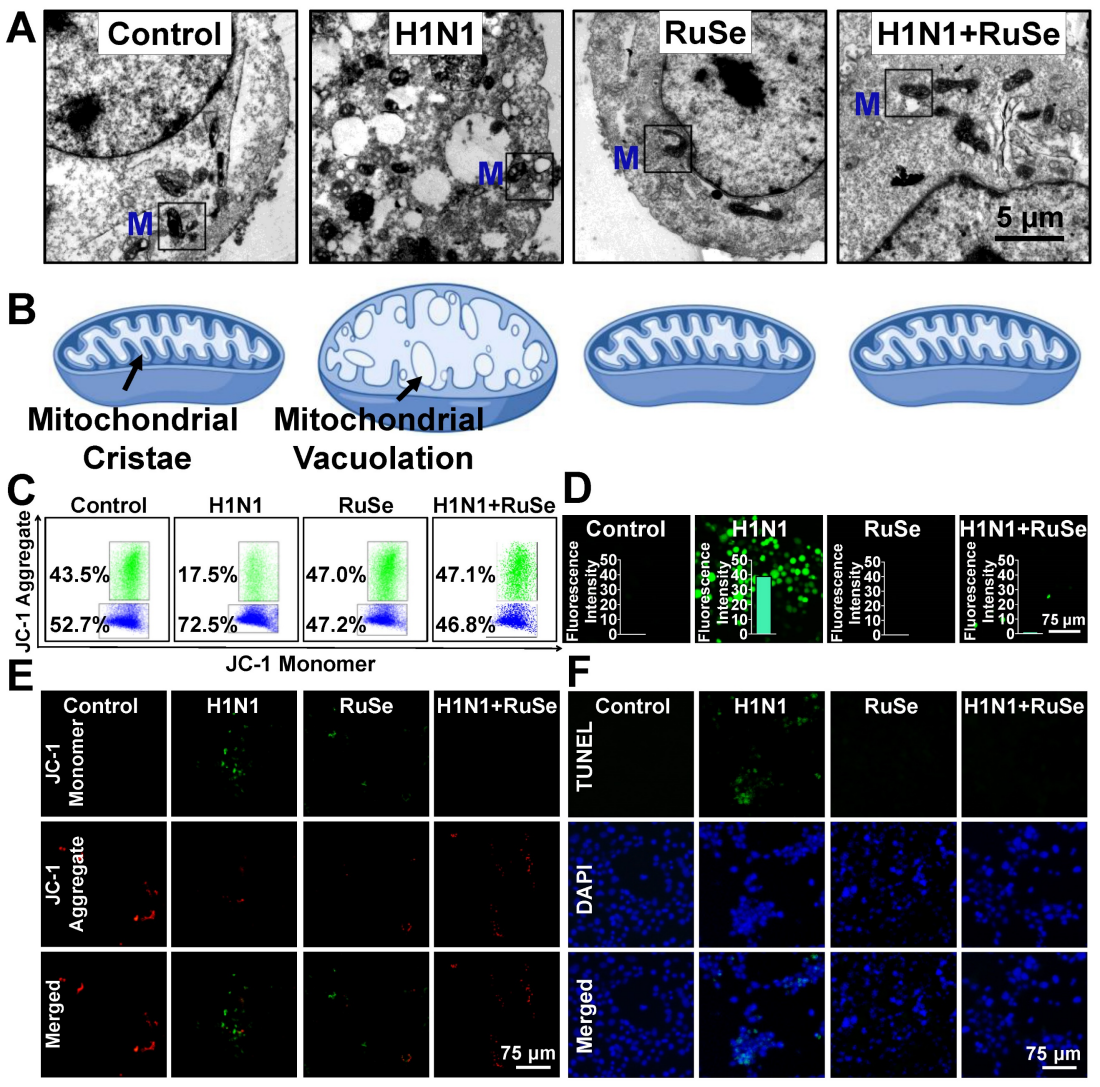
** RuSe inhibited cell apoptosis associated with H1N1-mediated mitochondrial oxidative damage.** (A) Electron microscopic sections of MDCK cells. The Control group was MDCK cells without treatment, the H1N1 group was H1N1 infected cells, the **RuSe** group was drug group, and the H1N1+RuSe group was treatment group. (B) Simulation of mitochondrial morphological changes in MDCK cells after treatment in each group. (C) and (D) Changes of mitochondrial membrane potential in H1N1-infected cells treated with **RuSe** by flow cytometry and fluorescence microscopy. (E) Intracellular mitochondria-mediated ROS generation was detected using the ROS probe DCFH-DA. (F) Detection of H1N1-mediated DNA fragmentation using TUNEL-DAPI staining. The scale in the figure is 75 μm. Each value represents means ± SD (n = 3).

The cell morphology under microscope suggested that H1N1-infected MCDK cells showed typical characteristics of apoptosis, such as round and small cells, pyknotic nuclei, and apoptotic vesicles. Similar changes were observed under electron microscopy. Chromatin agglutination, nucleolus fragmentation, cytoskeleton disintegration and apoptotic vesicles were observed in MDCK cells infected with influenza. Meanwhile, the mitochondria in the cell swelled, and the crest on the intima is absent, filling with vesicles (Figure 3A), which was consistent with the morphological characteristics of typical cell apoptosis and oxidative stress injury. Figure 3B is a simulation of the characteristics of intracellular mitochondria as shown in Figure 3A.

Based on morphological changes induced by influenza infection, such as vacuolation and mitochondrial swelling, it was reasonable to speculate that H1N1 induced apoptosis by causing mitochondrial damage and oxidative stress, which **RuSe** suppressed. To test this hypothesis, we examined changes in mitochondrial membrane potential, which is a milestone event in the early stage of apoptosis. It was found that, as shown in Figure 3C, the mitochondrial membrane potential (ratio of JC-1 polymer to monomer) of the cells infected with H1N1 decreased significantly, and that of H1N1+**RuSe** recovered to close to the normal group. The results of microscopic observation were consistent with those of flow examination. When the mitochondrial membrane potential is high, JC-1 aggregates in the matrix of mitochondria to form a polymer and produce red fluorescence; otherwise, it produces green fluorescence. As shown in Figure 3D, the green fluorescence of the H1N1 group was significantly enhanced, while the green fluorescence of H1N1+**RuSe** was weakened and the red fluorescence was enhanced, indicating **RuSe** inhibited the decline of mitochondrial membrane potential mediated by H1N1. Generally, reduction of mitochondrial membrane potential leads to the accumulation of reactive oxygen species (ROS), which associated with damage to intracellular macromolecules (such as proteins and nucleic acids) 26. As shown in Figure 3E, ROS accumulation in different treatment groups was detected by DCFA probe, and the results showed that ROS accumulation increased in cells infected with H1N1 and decreased after **RuSe** treatment. Histogram showed the result of quantization of green fluorescence intensity in the image by Image J software. On the other hand, ROS accumulation leads to the breakdown of intracellular nucleic acid (DNA) and even apoptosis 27. TUNEL probe can label terminal transferase and accurately reflect the characteristics of apoptotic cells. The features of nuclear condensation and DNA fragmentation of cells infected with H1N1 were shown in Figure 3F. Treatment with **RuSe** remarkably changed nuclear morphology induced by H1N1. These results suggested that **RuSe** inhibited mitochondrial damage induced by H1N1, reduced intracellular ROS accumulation and prevented cell apoptosis. Combined with the results in Figure 3, **RuSe** inhibited the apoptosis associated with mitochondrial oxidative damage mediated by H1N1 in MDCK cells.

### es H1N1-mediated lung apoptosis and lung inflammation in mice

**Figure 4:**
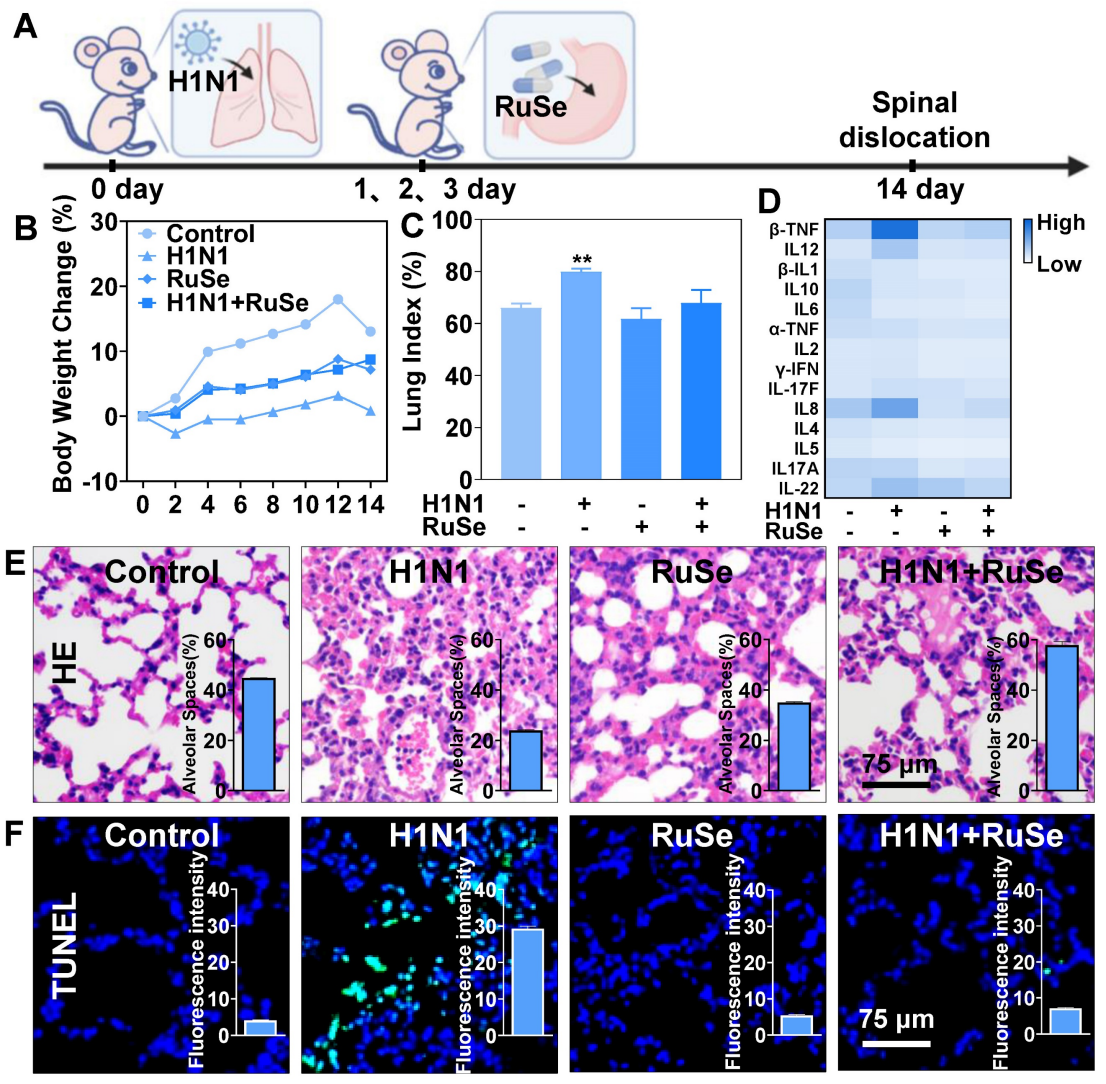
** RuSe suppressed H1N1-mediated pneumonia and lung tissue apoptosis in mice.** (A) BALB/c mice were treated intragastrically with 2.5 Mm **RuSe** after intranasal infusion of 1000 TCID_50_/mL H1N1. (B) After infection with H1N1, mice were treated with **RuSe** and weight changes were recorded. (C) The difference of lung index in different treatment groups (lung weight/body weight x 100%). (D) Blood samples were collected on the 14^th^ day after H1N1 infection of mice in different treatment groups, and the changes of serum cytokines were detected by flow cytometry. (E) After 14 days of different treatments, the lung tissues of mice were taken out, and the morphological changes of the lung tissues were observed by HE staining. (F) After TUNEL-DAPI staining, the lung tissue apoptosis of mice was observed under microscope. (Each value represents the mean ± SD (n = 3).

As mentioned above, it was suggested that **RuSe** can inhibit cell apoptosis induced by H1N1 *in vitro*, and we verified that **RuSe** played a similar role in mice. Figure 4A showed a diagram of infection and treatment in mice. After the mice were infected with H1N1 nasal drop, **RuSe** was used for 3 consecutive days of intragastric administration, and the mice were sacrificed for relevant tests at 14 days. The changes in body weight of mice are shown in Figure 4B. On the second day after infection, the weight of mice in the H1N1 virus-infected group decreased significantly, and the weight of mice in the H1N1 virus-infected group did not increase until day 14. Compared with the weight gain of normal mice, it showed that the mice with H1N1 infection had more serious lung inflammation, less food intake and no weight gain. The weight of mice in **RuSe** treatment group increased significantly within 14 days, indicating that **RuSe** has a certain therapeutic effect on pneumonia caused by influenza. Influenza infection causesd lung inflammation in mice, and the exudation of inflammatory factors exacerbated pneumonia 28. The lung index value objectively indicates the degree of pneumonia 29. Lung index of mice was calculated for each group (Figure 4C): control (0.65), H1N1 (0.76), **RuSe** (0.66) and H1N1+ **RuSe** (0.71). These results indicated that influenza infection leads to severe lung inflammatory exudation in mice, and **RuSe** treatment effectively alleviates the inflammatory exudation.

On the other hand, flu-induced high expression of cytokines can aggravate the severity of pneumonia. Inflammatory cytokine storm is an important manifestation of H1N1-mediated pneumonia 30. TNF-β mediates many inflammatory, immune stimulation and antiviral responses. IL-8 is a cytokine secreted by Th1 cells, which mainly mediates the production of adjuvant antibodies in the immune response related to cytotoxicity and local inflammation. IL-12 May stimulate the growth of immune factors. IL-22 promotes survival primarily by inducing epithelial cell proliferation and inhibiting apoptosis. In addition, Interleukin-22 has been shown to have lung epithelial protection against bacterial and viral pathogens at the mucosal immune interface. IL-17 is a pro-inflammatory cytokine produced by Th17 cells and macrophages, etc. When autoimmune disorders occur, the pro-inflammatory effect of IL-17 can lead to pathogenic inflammation 31. We also investigated the effect of inflammatory factors on pneumonia and found that H1N1 infection led to the increase of pro-inflammatory factors β-TNF, IL-8, IL-12, IL-22, and IL-17, and the levels of inflammatory factors returned to normal after **RuSe** treatment (Figure 4D). These results suggested that **RuSe** alleviated lung tissue damage and inhibited the virus by inhibiting the inflammatory response induced by influenza. Then, we conducted morphological detection on the lung tissues of mice, such as hematoxylin-Eosin (HE) staining and TUNEL-DAPI staining. Compared with the normal group, the lungs in the viral group were congested and edematous with a dark brown appearance. The lung morphology of H1N1-infected mice was similar to the lung biopsies of H1N1-infected patients 32. As shown in Figure 4E, HE staining results showed that the alveoli of mice infected with H1N1 collapsed, and edema appeared around blood vessels and bronchi. In addition, normal tissue structures such as alveoli and bronchioles disappeared, and a large number of inflammatory cells infiltrated in the alveoli. While **RuSe** significantly reduced the pathological features of lung tissue.

The histogram in Figure 4E showed the number of alveoli in lung tissue sections (calculated by IMAGE J), showing a sharp decrease in the number of alveoli in mice infected with H1N1, but not in mice treated with **RuSe**. Moreover, ROS attacks cause damage to intracellular biological macromolecules, such as DNA disruption and protein inactivation 33. TUNEL-DPAI staining results (Figure 4F) showed nucleic acid fragmentation and nuclear pyrosis in lung tissues of mice infected with H1N1, indicating that **RuSe** alleviated lung tissue damage by inhibiting ROS, which was consistent with experimental results at the cell level. The histogram in Figure 4F showed the intensity of green fluorescence. It is apparent that the green fluorescence in the H1N1 group was the strongest and the nucleic acid breakage was the most serious, while the H1N1+**RuSe** group was similar to the control. Combined with the results of the above experiments, **RuSe** alleviated virus-mediated lung parenchyma and higher inflammation level by inhibiting lung tissue apoptosis.

### RuSe inhibits H1N1-mediated apoptosis *in vivo* and *in vitro* by regulating proteins associated with the apoptotic pathway

**Figure 5:**
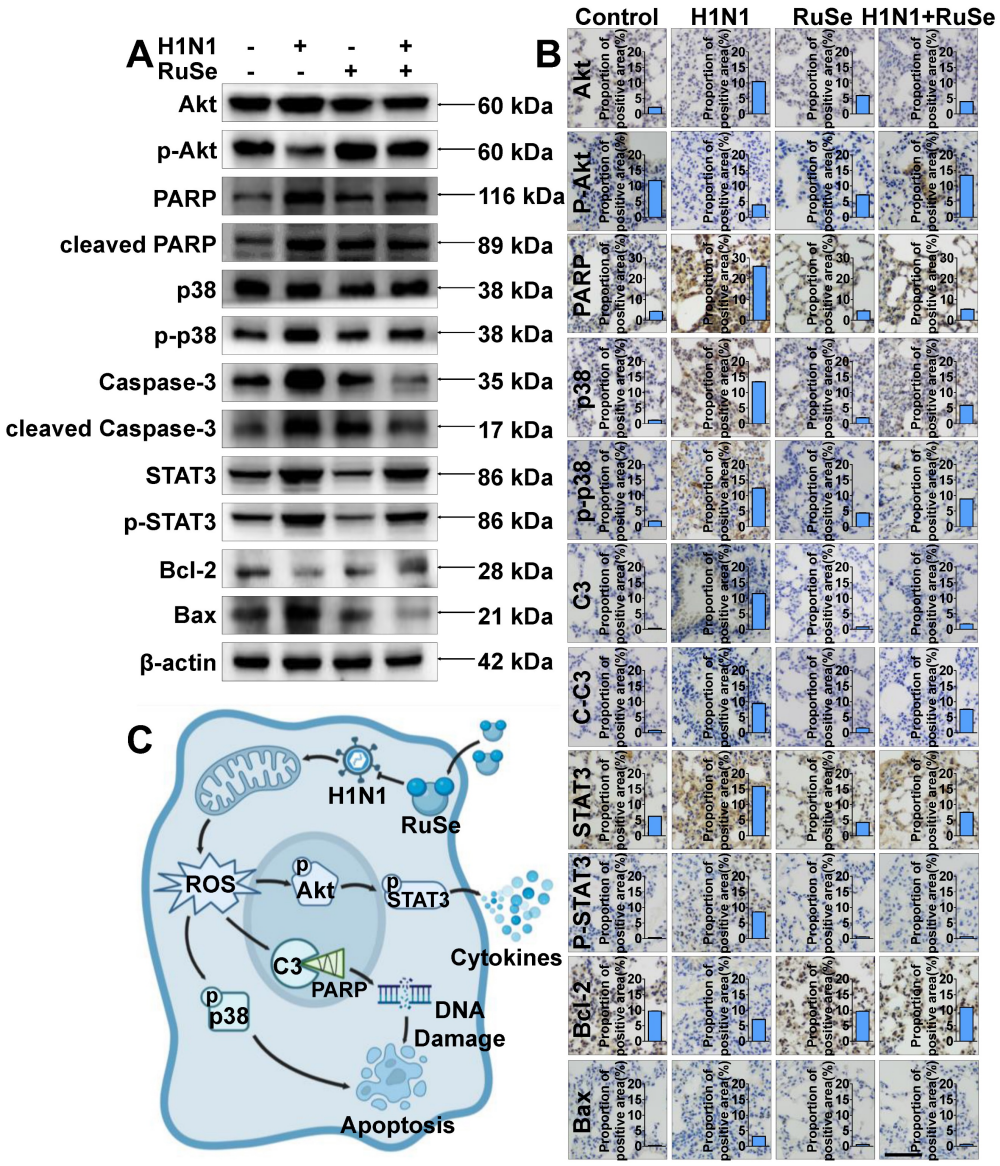
** RuSe inhibits H1N1-mediated apoptosis by regulating proteins associated with the apoptosis pathway.** (A) After 48 h of different treatment of MDCK cells, western blotting was performed on the proteins of the lysed cells to observe the regulation of apoptotic proteins by **RuSe**. (B) The expression of apoptosis-related proteins in the lungs of mice infected with H1N1 was measured by immunohistochemistry after lung tissue slices were taken after different treatments. (C) Schematic illustration of **RuSe** regulating the expression of intracellular apoptosis-related proteins and inhibiting virus-mediated apoptosis after H1N1 infection. Each value represents the mean ±SD (n = 3).

Akt, also known as protein kinase B (PKB), is a serine/threonine kinase. Activated Akt phosphorylates target proteins on cell membranes, whose phosphorylation stimulates cell survival, growth, and proliferation 34. As shown in Figure 5A, the phosphorylation of Akt was reduced after H1N1 virus infection, and the expression of p-Akt was up-regulated after **RuSe** treatment. Meanwhile, the expression of p-STAT3 showed an opposite trend to that of p-Akt. H1N1 inhibited Akt phosphorylation and in turn promoted STAT3 phosphorylation. Signal transduction and transcriptional activator 3 (STAT3) belongs to a family of proteins known as transcription factors that are critical for regulating the cell cycle and immune response. Activation of STAT3 is achieved by phosphorylation 35. Many reports have confirmed the effect of oxidative stress on the STAT3 cascade, such as increased phosphorylation of STAT3 and up-regulation of its DNA binding activity after different oxidative stimuli 36. On the other hand, STAT3 plays a major role in intracellular signal transduction of immune cells. It transduces extracellular signals transmitted by a large number of cytokines, lymphatic factors and growth factors, and participates in the process of amplifying cytokine expression 37. As shown in Figure 5A and Figure 5C, STAT3 activated by phosphorylation mediates the transcription of many pro-inflammatory cytokines, leading to inflammatory responses in cells. As shown in Figure 5A, the expression trends of STAT3 and Akt in mouse lung tissues were consistent with those *in vitro* experiments (Figure 5B). In conclusion, **RuSe** may regulate the activation of STAT3 by increasing the expression of p-Akt, thus playing a role in regulating cellular inflammatory response. Caspase protein family Caspase-3 (cysteine proteinase protein 3) is responsible for the hydrolysis and cleavage of various proteins and is an essential executor of apoptosis 38. The expression and activation of Caspase is an important step of cell apoptosis. PARP is a substrate for Caspase-3 that induces cell survival by repairing DNA. During apoptosis, PARP is cleaved into two fragments by Caspase-3, resulting in protein inactivation. The activity of endonuclease affected by the negative regulation of PARP was increased, and the DNA between nucleosomes was lysed and apoptosis was induced 39. As shown in Figure 5A, the expression of Caspase-3 protein increased after H1N1 virus infection and decreased after **RuSe** treatment, and the expression trend of PARP was consistent with Caspase-3. Meanwhile, the expression trends of Caspase-3 and PARP in mouse lung tissues were consistent with *in vitro* experiments (Figure 5A). In conclusion, **RuSe** may inhibit the activity of PARP by reducing the expression of Caspase-3 and other apoptotic executive proteins, thereby regulating and alleviating the damage of internal DNA, and thereby regulating the process of apoptosis.

P38 mitogen-activated protein kinase (p38 MAPK) is a stress-activated protein kinase, which is closely related to the initiation of apoptosis and the rest of the cell cycle. Specifically, p38 can mediate the phosphorylation of the anti-apoptotic Bcl-2 protein, leading to ubiquitination and degradation of Bcl-2, which leads to cell death 40. Bcl-2 gene (B cell lymphoma/leukemia-2 gene) is an oncogene, which has an obvious inhibitory effect on apoptosis 41. As shown in Figure 5A, the expression of p38 protein increased after H1N1 virus infection, decreased after **RuSe** treatment, and the expression trend of Bcl-2 was opposite to p38, while the expression trend of bax was consistent with p38. In addition, as shown in Figure 5B, the expression trends of p38, Bcl-2 and Akt in mouse lung tissues were consistent with those *in vitro* experiments (Figure 5A). In conclusion, **RuSe** may inhibit the degradation of Bcl-2 by reducing the expression of p38 protein kinase, thus inhibiting the process of regulating cell apoptosis. Combined with the results of *in vitro* western blotting and *in vivo* immunohistochemistry of lung tissue, **RuSe** inhibited apoptosis by regulating the expression of apoptosis-related proteins.

To further investigate the anti-influenza mechanism of **RuSe** *in vivo*, immunohistochemical assays were used to verify the apoptotic proteins regulated by **RuSe**. Immunohistochemistry revealed that **RuSe** affected the apoptotic process of influenza-infected lung tissues by regulating the expression of apoptotic proteins, as shown in Figure 5B. Consistent with the expression trend in cell experiments, **RuSe** inhibited H1N1-mediated apoptosis by down-regulating the expression and activation of PARP, p38, Caspase-3, STAT3 and Bax. On the other hand, **RuSe** inhibited H1N1-mediated apoptosis by up regulating the expression and activation of Akt and Bcl-2. Combining the results of immunohistochemistry and *in vitro* experiments, **RuSe** regulates apoptosis *in vivo* and *in vitro* by regulating ROS, an inflammatory apoptotic protein.

### RuSe inhibits H1N1-mediated inflammation by regulating selenium content and selenoprotein expression in mice

Selenium is an essential nutrient found in the human body in the form of selenocysteine and selenomethionine. Selenoproteins are anti-inflammatory antioxidant fighters that protect healthy cells from oxidative damage induced by reactive oxygen species. After ingestion, **RuSe** is absorbed and transported from intestinal tract to liver to synthesize selenomethionine (SeMet), which is stored in the methionine pool for further synthesis of selenomethionine 42. The final step in selenium metabolism is the conversion of SeMet to Sec through the sulfur-conversion pathway, followed by selenoprotein biosynthesis 43. Selenocysteine is the bioactive center of selenoprotein and the basis of selenoprotein's antioxidant function. The metabolism of selenocysteine is positively correlated with the content and activity of selenoprotein. In this regard, we further explored the total selenium, selenoprotein content and selenium metabolism of **RuSe** in mice, so as to determine whether selenoprotein plays a role in alleviating virus-mediated oxidative damage.

**Figure 6:**
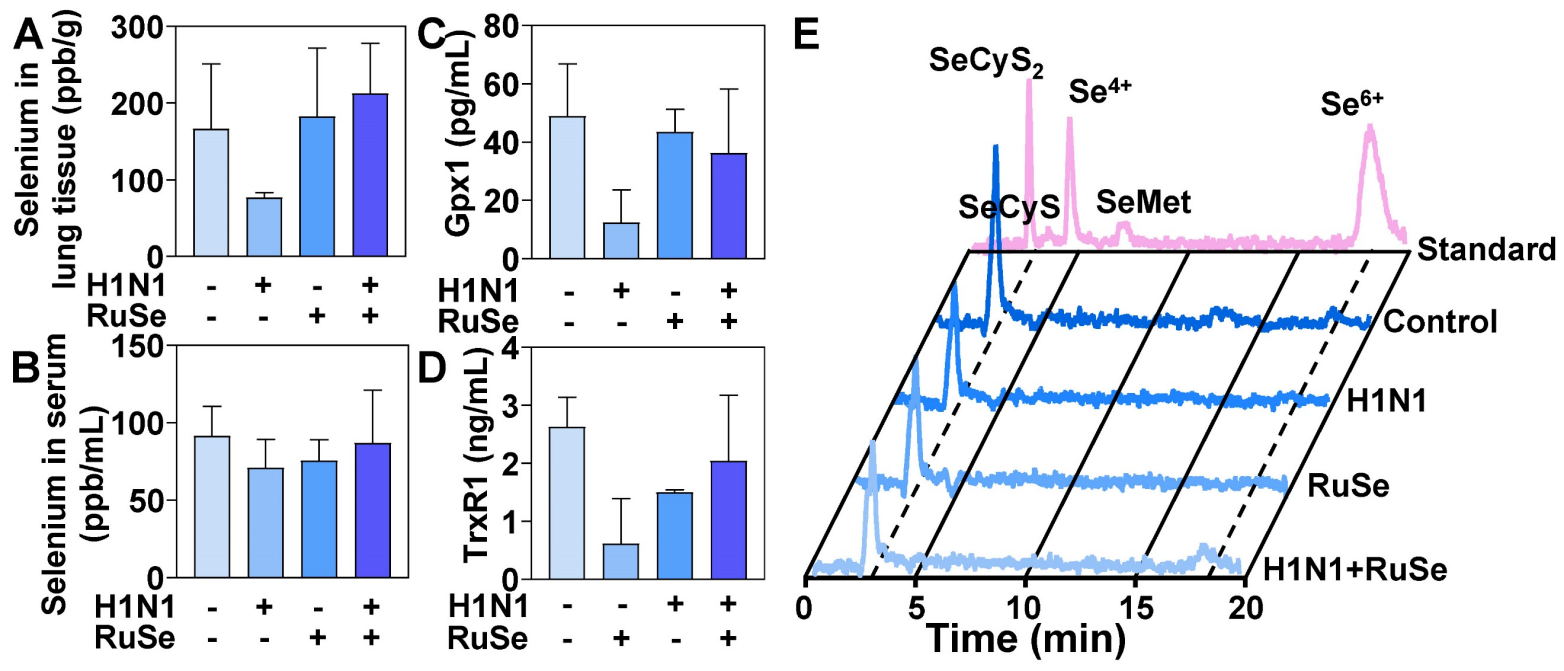
** RuSe inhibited virus-mediated inflammatory response by regulating selenium content and selenoprotein changes in mice.** (A) Determination of total selenium in mouse lung tissue by ICP-MS. (B) Determination of total selenium in mouse serum by ICP-MS. (C) Determination of Gpx1 in mouse serum by ELISA. (D) The content of TrxR1 in serum of mice was determined by ELISA. (E) Serum selenium metabolism in mice infected with H1N1 after different treatments. Each value represents means ± SD (n = 3).

As shown in Figure 6A, H1N1 infection resulted in a decrease in the total amount of selenium in lung tissues of BALB/C mice. After treatment with **RuSe**, the H1N1+**RuSe** group significantly recovered. These results indicated that **RuSe** increased the selenium content in lung tissue, enhance the antioxidant capacity of lung tissue, and alleviate the oxidative damage of lung tissue mediated by virus. H1N1 infection leaded to decreased selenium levels in lung tissue, which may be associated with an excessive oxidative stress response and inflammation. While **RuSe** increased the selenium content of lung tissue and relieved the oxidative stress-related lung injury caused by H1N1. As shown in Figure 6B, the serum selenium content of mice decreased due to viral infection, and the serum selenium level returned to normal after **RuSe** treatment, which was similar to the results of lung tissue. On the other hand, GPx1 and TrxR1 mainly perform antioxidant function in organisms, which can alleviate oxidative damage mediated by influenza. The changes of total selenoproteins affected the expression of GPx1 and TrxR1. As shown in Figure 6C, the serum GPx1 content of mice in the virus infection group decreased significantly, and the GPx1 content increased significantly after **RuSe** treatment. As shown in Figure 6D, the serum TrxR1 content of mice in the H1N1 infection group decreased significantly, and the TrxR1 content recovered to a certain extent after **RuSe** treatment. These results indicate that in influenza infected mice, the decrease of total selenium content leads to the decrease of GPx1, TrxR1 and other selenoproteins expression, the overall decrease of antioxidant capacity, the high level of inflammation, and the obvious symptoms of pneumonia. The supplementation of **RuSe** can improve the selenium content in the body, thereby enhancing the level of selenoprotein, improving the antioxidant function of the body, and leading to the decrease of inflammation. The metabolism of selenium element in Figure 6E verifies the reason for the increased expression of selenoprotein. The level of SeCyS_2_ metabolism was decreased in the viral infection group compared with the control group. SeCyS_2_ is selenocysteine and can be interconverted with selenocysteine (SeCyS), which is the active center of selenoprotein. Selenium metabolism in the **RuSe** treatment group was dominated by SeCyS_2_, and the conversion between SeCyS_2_ and SeCyS resulted in the increase of selenoprotein active centers (SeCyS) and selenoprotein content. Based on the results in Figure 6, **RuSe** increases the content of selenium in the body and is mainly metabolized into SeCyS_2_, which acts as the active center of selenoprotein. The expression level of selenoprotein is increased, and the antioxidant ability is improved, so the inflammatory symptoms are alleviated.

### RuSe alleviates pneumonia and lung cell apoptosis in selenium-deficient mice infected with H1N1

**Figure 7:**
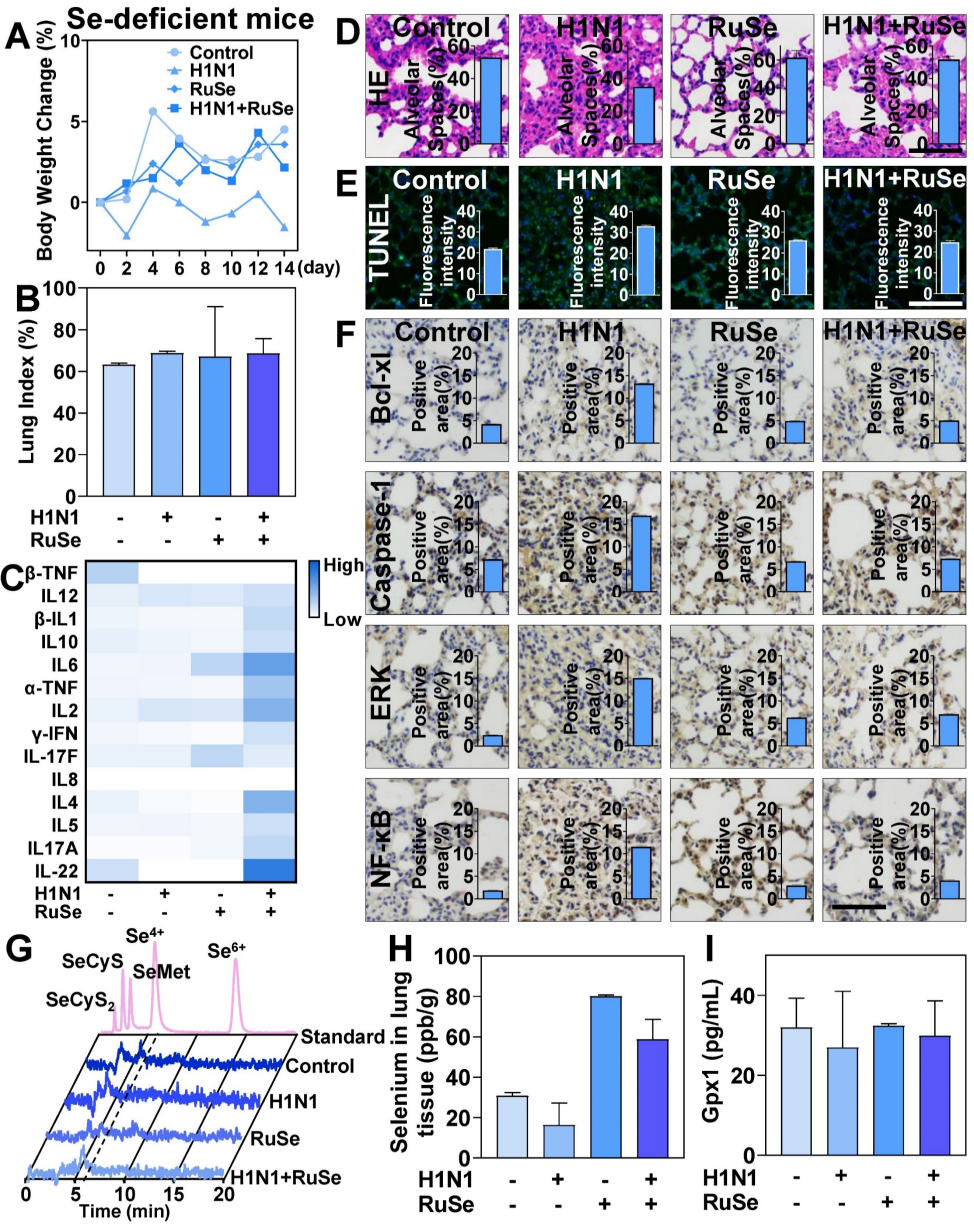
** RuSe inhibited lung inflammation and apoptosis in selenium-deficient mice infected with H1N1.** (A) Body weight changes in selenium-deficient mice infected with H1N1 after **RuSe** treatment. (B) Differences in lung index in selenium-deficient mice infected with H1N1 after **RuSe** treatment. (C) The corresponding cytokines in selenium-deficient mice infected with H1N1 after **RuSe** treatment. (D) HE staining was used to observe the morphological changes in the lungs of selenium-deficient mice infected with H1N1 after **RuSe** treatment. (E) TUNEL staining was used to detect the degree of DNA fragmentation in lung tissue of **RuSe**-treated mice infected with influenza. (F) Immunohistochemistry was used to determine the signaling pathways regulated by H1N1 infection in selenium-deficient mice. (G) Selenium metabolism in blood of selenium-deficient mice infected with influenza virus treated with **RuSe** on day 14. (H) Changes of selenium content in lung tissue of mice in each group on the 14^th^ day after infection. (I) The level of GPx1 in the blood of selenium-deficient mice at day 14 after infection with influenza virus was detected with ELISA. The scale in the figure is 75 μm. Each value represents means ± SD (n = 3).

To further investigate the role of selenium in influenza virus infection, we measured changes in a selenium-deficient mouse model treated with **RuSe**. The weight changes of Se-deficient mice were shown in Figure 7A. The weight of Se-deficient mice increased steadily after 14 days of feeding, while the weight of Se-deficient mice infected with virus did not increase significantly. Lung index is an important index to measure lung parenchyma. The changes of lung index of Se-deficient mice were shown in Figure 7B. The lung index of Se-deficient mice infected with virus increased significantly, indicating that virus infection caused severe lung damage to Se-deficient mice. On the other hand, selenium deficiency causes the body to reduce immune function and immune response ability. Lymphocytes and the immune system exhibit some functional changes with selenium deficiency and selenium replacement. Members of the selenoprotein family that have been shown to be associated with lymphocyte activity and play a general role in MHC-dependent presentation are selenoproteins and are responsible for immune dysfunction in Se deficiency 44. The immune response of mice was shown in Figure 7C, and the immune response of selenium-deficient mice was significantly weakened. Despite H1N1 infection, the immune response of selenium-deficient mice was not mobilized, and the absolute values of serum cytokines in mice were low.

After the treatment with **RuSe**, the immune ability of mice was restored to some extent. In addition, the lung tissue infection of selenium-deficient mice was also observed from the aspect of lung morphology. As shown in Figure 7D and Figure 7E, after infection with H1N1 in se-deficient mice, the lung morphology changed significantly. In the H1N1 infected group, the alveolar space was reduced, the alveolar structure collapsed, and the pulmonary inflammatory cells infiltrated. After **RuSe** treatment, the lung morphology of selenium-deficient H1N1 infected mice was not significantly different from that of the control group. These data suggested that **RuSe** is helpful in inhibiting lung tissue inflammation and fibrosis in selenium-deficient mice infected with influenza. At the same time, the lung tissue apoptosis of mice was significantly increased in H1N1 infection with selenium deficiency and decreased after **RuSe** treatment. It is suggested that **RuSe** can reduce cell apoptosis in selenium-deficient mice infected with H1N1. Numerous studies have demonstrated that apoptosis of alveolar type II epithelial cell (AEC-II) plays a vital role in the process of pulmonary pneumonia and pulmonary fibrosis. Excessive apoptosis of AEC- I will lead to decrease in the number of structural cells and aggravate the pathological changes of lung tissue 45. Equally important, influenza infection regulates apoptotic regulatory proteins in mouse lung tissue. Anti-apoptotic Bcl-2 family proteins (such as Bcl-xL) bind to Bax and Bak to inactivate them and prevent mitochondrial permeation. As shown in Figure 7F, influenza virus upregulation of Bcl-xL to influence cell motility is consistent with previous studies. Caspase-1 is an inflammatory Caspase involved in apoptotic cell death and the release of inflammatory cytokines in response to pathogen patterns and endogenous stimulators. In the lungs of selenium-deficient mice infected with H1N1, the expression of Caspase-1 was significantly increased and appropriately decreased after **RuSe** treatment. It has been shown that ERK1/2 kinase has proapoptotic function under certain conditions, and enhanced ERK1/2 signaling can lead to cell death 46. This is consistent with the results of the present study, which showed that the expression of ERK was increased in selenium-deficient mice infected with H1N1 and promoted apoptosis of lung tissue cells in mice, while the expression of ERK was appropriately decreased after treatment with **RuSe**. In addition, the NF-κB family and its mediated cell signal transduction play an important role in apoptosis. It is involved in the transcriptional regulation of many apoptosis-related genes, and has a bidirectional effect of inhibiting and promoting apoptosis 47. As shown in Figure 7F, the expression of NF-κB increased in the lung tissue of virus-infected mice, but decreased after **RuSe** treatment. Synthesize the results above, **RuSe** regulates apoptosis-related and inflammation-related proteins in mouse lung tissue, thereby regulating lung tissue inflammation and fibrosis. The blood selenium metabolism of selenium-deficient mice after **RuSe** supplementation was shown in Figure 7G. Se^4+^ metabolism decreased significantly after infection with H1N1 in selenium-deficient mice, and Se^4+^ increased significantly after treatment with **RuSe**. Similarly, selenium content in lung tissues of selenium-deficient mice infected with H1N1 decreased. After treatment with **RuSe**, selenium content in lung tissues of the treatment group increased significantly, as shown in Figure 7H. In addition, the results in Figure 7I also suggested that **RuSe** can also increase the serum GPx1 content of selenium-deficient mice in response to oxidative stress damage caused by influenza infection.

## Conclusions

A novel antiinfluenza drug **RuSe** was prepared by using ruthenium and selenium with low toxicity and high absorption efficiency with antiviral activity. The antagonistic effect of **RuSe** on influenza virus was described from three aspects. First, **RuSe** inhibited viral influenza virus-mediated mitochondria-related apoptosis. *In vivo* experiments, **RuSe** inhibited membrane phosphatidylserine eversion, mitochondrial membrane potential decline, mitochondrial crest remodeling and cavitation. More importantly, **RuSe** inhibited ROS accumulation and DNA fragmentation caused by mitochondrial damage. *In vivo* studies, **RuSe** has been shown to inhibit influenza-mediated pneumonia and apoptosis-associated pulmonary fibrosis in AEC-II. The low-toxicity **RuSe** saved weight loss in mice infected with H1N1 and inhibited infection-induced lung parenchyma and over activation of inflammatory factors. In addition, **RuSe** regulated the expression of apoptotic protein in mouse lung tissue, affected the apoptotic process of AEC-II, and inhibited the pulmonary fibrosis associated with AEC-II apoptosis. Secondly, **RuSe** played a certain direct antiviral effect *in vitro*. **RuSe** has certain inhibitory effects on the virulence, nucleic acid replication, NA activity and influenza protein expression of H1N1. Immunofluorescence assay showed that **RuSe** had a significant anti-influenza effect, which inhibited the nucleation of NP and reduced the co-localization of NP in the cytoplasm of influenza virus. In addition, **RuSe** supplementation mitigated the decreased selenium levels in lung tissue and serum of mice infected with influenza virus. When **RuSe** was ingested by the intestinal tract of mice, the selenium content of the body was increased, and the selenium element was metabolized into selenocysteine (SeCyS_2_). SeCyS_2_ was the active center of selenoproteins, there by the expression of selenoproteins such as GPx1 and TrxR1 was increased. With the increase of the expression of the antioxidant enzymes GPx1 and TrxR1, apoptosis associated with influenza-mediated oxidative damage in lung tissue was also mitigated. Combined with the above results, **RuSe** showed antiviral effects in three aspects: inhibition of influenza-mediated apoptosis and inflammation, direct inhibition of replication and protein synthesis of H1N1 as well as increase of selenium content and selenium protein in mice, thus antagonizing virus-mediated oxidative damage. After a more thorough evaluation, **RuSe** has the potential to be a very promising anti-influenza drug.

## Supplementary Material

Supplementary method and table.

## Abbreviations

HAV: Influenza A
RuSe: Ru(biim)(PhenSe)_2_
ROS: reactive oxygen species
MDCK: Madin-Darby canine kidney
ATCC: American Type Culture Collection
FBS: Fetal bovine serum
DMEM: Dulbecco's modified Eagle's medium
CST: Cell Signaling Technology
NA: Neuraminidase
PI: Propidium iodide
DCF-DA: 2',7'-dichlorofluorescein diacetate
ECL: Enhanced Chemiluminescence
TCID_50_: median tissue culture infective dose
