# Supplementary material for: Selenium-ruthenium complex blocks H1N1 influenza virus-induced cell damage by activating GPx1/TrxR1
Source: Theranostics. 2023 Mar 21;13(6):1843–59. doi: 10.7150/thno.83522 (PMC10091872; doi:10.7150/thno.83522)
Supplement: Supplementary file 1 — Supplementary method and table. [file thnov13p1843s1.pdf]

Supporting information

For

Selenium-ruthenium complex blocks H1N1 influenza virus-induced cell damage by activating GPx1/TrxR1

Yinghua Li[1], Danyang Chen[1], Jingyao Su[1], Mingkai Chen[2], Tianfeng Chen[2]✉, Wei Jia[3]✉ and Bing Zhu[1]✉

[1] *Center Laboratory, Guangzhou Women and Children's Medical Center, Guangzhou Medical University, Guangzhou 510120, China*

[2] *Department of Chemistry, Jinan University, Guangzhou 510632, China*

[3] *Department of Pediatric Urology, Guangzhou Women and Children's Medical Center, Guangzhou Medical University, Guangzhou 510120, China*

1 Calculation of the titer of influenza virus

$TCID_{50}$ represents the amount of virus that causes cytopathic effects in 50% of infected cells, and can be used to estimate the intensity and content of viral infection. The brief calculation method is as follows. MDCK cells were uniformly inoculated into 96-well plates with a cell density of $8*10^4$/ mL and placed in an incubator at 37 °C overnight. The virus culture solution was used to dilute the original virus continuously 10-fold, from 10^{-1} to 10^{-12}. The diluted virus was inoculated into 96-well culture plates, and each well was inoculated with 100 μL. After incubating at 33 °C for 2 hours, the virus diluent was discarded and 100 μL virus culture medium was added into the incubator at 33 °C. The results were observed daily and recorded, usually for 5-7 days until the cytopathic condition was stable. $TCID_{50}$ was calculated by Reed-Muench assay. The results were recorded and calculated as Table 1.

Table 1 Titer of H1N1

Dilution of H1N1	CPE	None CPE	Accumulation		The ratio of CPE (%)
			CPE	None CPE	
10^{-1}	8	0	43	0	100(43/43)
10^{-2}	8	0	35	0	100(35/35)
10^{-3}	8	0	27	0	100(27/27)
10^{-4}	6	2	19	2	90(19/21)
10^{-5}	5	3	13	5	72(13/18)
10^{-6}	3	5	8	10	44(8/18)
10^{-7}	3	5	5	15	25(5/20)
10^{-8}	2	6	2	21	8(2/23)
10^{-9}	0	8	0	29	0(0/29)
10^{-10}	0	8	0	37	0(0/37)
10^{-11}	0	8	0	45	0(0/45)
10^{-12}	0	8	0	53	0(0/53)

The Reed - Muench assay was used to test the titer of H1N1. ($TCID_{50}=10^{-5.79}$)
